# *Helicobacter pylori* and gastrointestinal symptoms in diagnostics and adjuvant chemotherapy of colorectal cancer

**DOI:** 10.3892/ol.2013.1714

**Published:** 2013-11-29

**Authors:** LEENA-MAIJA SOVERI, PIA ÖSTERLUND, TARJA RUOTSALAINEN, TUIJA POUSSA, HILPI RAUTELIN, PETRI BONO

**Affiliations:** 1 Department of Oncology, Helsinki University Central Hospital, Helsinki 00029, Finland; 2 Clinical Department, University of Helsinki, Helsinki 00014, Finland; 3 STAT-Consulting, Nokia 37130, Finland; 4 Department of Bacteriology and Immunology, Haartman Institute, University of Helsinki, Helsinki 00014, Finland; 5 HUSLAB, Helsinki University Central Hospital Laboratory, Helsinki 00029, Finland; 6 Uppsala University, Department of Medical Sciences, Uppsala 75185, Sweden

**Keywords:** adjuvant treatment, colorectal cancer, gastrointestinal symptoms, *Helicobacter pylori*

## Abstract

5-Fluorouracil (5-FU)-based chemotherapy is the mainstay of adjuvant treatment for colorectal cancer (CRC). Few studies have explored *Helicobacter pylori* (*H. pylori*)-associated gastrointestinal symptoms in the diagnosis of CRC, and the association between *H. pylori* infection and gastrointestinal toxicity during adjuvant chemotherapy in CRC. Seventy-nine CRC patients were randomised in a prospective clinical trial to receive 5-FU and leucovorin administered as bolus injection (Mayo regimen) or continuous infusion (simplified de Gramont regimen). *H. pylori* antibodies were analysed at baseline, twice monthly during treatment and after treatment up to 12 months. Thirty-seven patients (47%) were *H. pylori-*seronegative at baseline. There was no significant association between baseline *H. pylori* seropositivity (n=42; 53%) and oro-gastrointestinal toxicity during chemotherapy. The median time from symptom onset of CRC to surgery was significantly longer in patients with *H. pylori* infection (median time, 6 vs. 5 months; P=0.012). Functional dyspeptic symptoms at presentation significantly delayed diagnosis (median time, 7.5 vs. 5 months; P=0.035), whereas anaemia, bowel symptoms, occlusion, blood in the stool, infection and hypolactasia did not. We conclude that there is no association between *H. pylori* status and gastrointestinal toxicity in CRC patients during chemotherapy. Dyspeptic symptoms and presence of *H. pylori* may delay the diagnosis of CRC. (www.controlled-trials.com/ISRCTN98405441).

## Introduction

Gastrointestinal symptoms including heartburn, flatulence and changes in bowel habits are extremely common complaints. In the majority of cases, a benign aetiology is responsible, but symptoms may also be associated with colorectal cancer (CRC). The mean time from symptom onset to diagnosis of CRC in Finnish patients is 10 months (1). The diagnostic delay is partly caused by patients being ashamed of their symptoms (91% of Finnish people), but most commonly due to the nonspecific and vague character of CRC symptoms (2–3). Work-up for abdominal complaints is often terminated at the diagnosis of common findings, including lactose intolerance, irritable bowel syndrome and *Helicobacter pylori* (*H. pylori*) infection.

*H. pylori* is a gram-negative bacterium, which colonizes the gastric epithelium and induces chronic inflammation of the gastric mucosa (4). *H. pylori* is the most important single risk factor for peptic ulcer disease (5). Infection is usually acquired in childhood and adolescence, (6–7) and tends to persist unless eradicated (7–8). Although prevalence of the infection in the western world has rapidly decreased during the past decades (7,9), at least half of the world’s population remains infected today (5). The majority of patients are asymptomatic, but in approximately 8% of *H. pylori*-positive dyspepsia patients, eradication of the bacterium leads to long-term relief of dyspepsia. Eradication is more effective than any other treatment and, therefore, dyspepsia remains an accepted indication for *H. pylori* eradication treatment (5).

Since 1994, *H. pylori* has been classified as a class I carcinogen (10). *H. pylori* is the most common proven risk factor for human non-cardiac gastric cancer, and eradication of *H. pylori* is the first-line therapy for low-grade gastric mucosa-associated lymphoid tissue lymphoma (5). Previous studies have evaluated the possible role of *H. pylori* in the development of CRC. However, large epidemiological studies remain missing and associations remain controversial (11–14). It has been proposed that *H. pylori* infection increases the secretion of gastrin, which may lead to mucosal cell alteration in the colorectum (12,14–15).

We hypothesised that *H. pylori* infection may influence tolerability of chemotherapy, and that eradication of the bacterium may be required to minimise chemotherapy-associated adverse events in CRC patients. As a secondary aim, we investigated whether *H. pylori*-associated symptoms were able to cause a delay in the diagnosis of CRC, and whether *H. pylori* status correlates with long-term outcome in CRC patients treated in a randomised prospective trial.

## Material and methods

This was an open, prospective, randomised single institution study in radically operated CRC patients. The LIPSYT trial is registered on http://www.controlled-trials.com/ISRCTN98405441. According to the study protocol, 80 patients with histologically proven CRC were included in the study. One patient did not start treatment due to a non-healing recto-vaginal fistula, leaving 79 patients for evaluation. The patients were treated at the Department of Oncology at Helsinki University Central Hospital (Helsinki, Finland), between November 1997 and October 1999. The minimum follow-up period of the surviving patients was 120 months.

Patients were eligible for inclusion if they were aged between 18 to 75 years, had histologically confirmed radically operated stage II–IV CRC (amendment to include radically operated stage IV patients in the tolerability part of the study was performed in December 1997), WHO performance status 0–2 and adequate bone marrow, kidney and liver function. Exclusion criteria included history of invasive cancer other than CRC; metabolic, neurological or psychiatric illness that was incompatible with chemotherapy; serious thromboembolic event currently under treatment; pregnancy, lactation or absence of adequate contraception in fertile patients.

The Ethical Review Board at Helsinki University Hospital approved the protocol and a written informed consent was obtained from all patients. No patients were lost to follow-up.

### Treatment regimens

Patients were randomised to receive postoperative adjuvant chemotherapy in CRC, combined with radiotherapy in rectal cancer if the distal margin of tumour was below the peritoneal fold. Randomisation was performed by minimization technique, with one out of six chances. Computer-based randomisation was performed by the oncologist (Pia Österlund) with gender, primary tumour and stage as factors. Adjuvant chemotherapy consisted of 5-FU and leucovorin (LV) as bolus injection (Mayo regimen) or continuous infusion (simplified de Gramont regimen) (16) according to randomisation. The Mayo regimen consisted of a 3- to 5-min intravenous bolus of 370–425 mg/m^2^ 5-FU and infusion of 10–20 mg/m^2^ LV on days 1–5 of a 4-week cycle, repeated six times. In rectal cancer during 5.5-week pelvic chemoradiation (50.4/1.8 Gy), starting on day 56, a single 500-mg/m^2^ 5-FU bolus was administered intravenously during days 1–3 on the first and fifth weeks. Radiation dose was based on CT planning and administered in three to four fields with high-energy photons. Following penetration of the preoperative Swedish radiotherapy results (17), certain patients received 25/5 Gy over 5 days preoperatively without concomitant chemotherapy. In these cases, postoperative adjuvant chemotherapy was provided. The de Gramont regimen consisted of a 2-h infusion of 200–400 mg/m^2^ LV followed by a 400-mg/m^2^ 5-FU bolus and continuous 3.0–3.6-g/m^2^ 5-FU infusion for 48 h, repeated every 14 days for 12 cycles. Rectal cancer patients received continuous infusion of 225 mg/m^2^ 5-FU during the same radiation treatment.

### Assessment of H. pylori status, lactose intolerance and treatment toxicity

Patients were evaluated at baseline prior to chemotherapy, every 4 weeks throughout chemotherapy and radiotherapy, and at 2–6 month intervals post-treatment up to five years and at 10 years. A complete medical history and physical examination were performed, including WHO performance status, height and weight, and laboratory assessment with tumour markers and blood cell counts.

Serum samples were collected prior to treatment, during the cancer therapy (at 2, 4 and 6 months) and after chemotherapy (at 8 and 12 months from initiation). Serum samples were stored at −20°C until analysis for *H. pylori* antibodies of the IgG and IgA classes using a locally validated in-house enzyme immunoassay with high sensitivity and specificity (18). *H. pylori* eradication was defined by ≥40% decline of the IgG titre within 6–12 months (9).

Seventy-seven patients were evaluated for lactose intolerance using an oral lactose tolerance test at baseline, and at 4 and 8 months after initiation of chemotherapy. The test was performed after a 12-h fast using an oral load of 50 g lactose, and blood glucose levels were measured three times at 20 min intervals and categorised as hypolactasia (a blood glucose level increase of <1.1 mmol/l), borderline finding (1.1–1.6 mmol/l) and normolactasia (>1.6 mmol/l).

Chemotherapy-associated toxicities were recorded in a patient diary and graded according to the NCI-C CTC version 2 (the Common Toxicity Criteria of the National Cancer Institute of Canada). The worst toxicity grade during adjuvant chemotherapy was taken into account in the analysis.

Presenting symptoms of CRC were assessed from hospital charts and by patient recall, and recorded into the patient diary at baseline. The severity of the symptoms at baseline as well as during the adjuvant treatment was evaluated.

All 79 patients were included in toxicity analysis. Six patients were excluded (n=73) in the analysis of diagnostic delay. In two patients it was impossible to determine the time from the onset of symptoms to diagnosis, one with gradually worsening anaemia and persistent constipation, the other with stomach pain and rectal bleeding from nonsymptomatic haemorrhoids. Four radically operated stage IV patients (local relapse or distant metastases resected) were included in the toxicity assessment, but excluded from diagnostic delay and survival analysis. Only stage II–III patients were included (n=73) in the efficacy analysis of disease-free survival and overall survival.

### Statistical analysis

The sample size calculation was based on the expected worst grade 3–4 oro-gastrointestinal toxicity. It was assumed that the toxicity was 60% in *H. pylori*-seropositive vs. 30% in *H. pylori*-seronegative patients. It was also assumed that there is no interaction between *H. pylori* positivity and chemotherapy treatment. With a 0.05 two-sided significance level and 80% power, the required sample size in each group is 40.

The gastrointestinal and dyspeptic symptoms during the chemotherapy treatment were the primary variables. The worst symptoms of grade 3–4 or symptoms of any grade, when appropriate, were compared between *H. pylori*-seropositive and -seronegative patients using the binary logistic regression analysis. As the patients were randomised to receive adjuvant chemotherapy (simplified de Gramont vs. Mayo regimen), the chemotherapy regimen was included as a categorical covariate. The interaction between *H. pylori* positivity and chemotherapy treatment was tested first, and if existence of interaction was not evident (P>0.10), the interaction term was omitted. The results are expressed as adjusted odds ratios with 95% confidence intervals (CIs). In case of interaction, the association between *H. pylori* and a symptom was assessed in both treatment groups separately.

Disease-free survival, overall survival and the diagnostic delay from onset of symptoms to surgery were the secondary variables. Disease-free survival was defined as the time from the date of initiation of the chemotherapy to the day of relapse, new CRC or mortality due to any cause. Overall survival was defined from the date of initiation of the chemotherapy to the date of mortality from any cause, or censored at the time of last follow-up, which was ≥10 years later. Cox regression analysis was applied to compare disease-free and overall survival between *H. pylori*-seropositive and -seronegative patients. The chemotherapy treatment was included as a categorical covariate and the interaction between *H. pylori* positivity. The results are given as adjusted hazard ratios with 95% CIs. The log-rank test was used to compare the diagnostic delay between *H. pylori*-seropositive and -seronegative patients.

The log-rank test was also used to compare the diagnostic delay between groups with lactose intolerance. Kaplan-Meier curves were drawn to describe the survival curves and diagnostic delay for *H. pylori*-seropositive and -seronegative patients. The χ^2^ and Mann-Whitney U tests were used for patient characteristics between *H. pylori*-seropositive vs. -seronegative patients. All tests were two-tailed and P<0.05 was considered to indicate a statistically significant result. IBM SPSS Statistics for Windows, version 20.0 (IBM Corp., Armonk, NY, USA) was used in calculations.

## Results

Patient characteristics are presented in Table I. The treatment arms were well-balanced by age, gender, and tumour stage and site. The median age of patients was 60 years (range, 31–76). At baseline 37 patients were *H. Pylori*-seropositive and 42 patients were *H. Pylori-*seronegative, of which two had received eradication treatment successfully years earlier. The 42 patients who were seronegative prior to chemotherapy remained negative for the next 12 months, and after chemotherapy. A total of 30 patients had positive *H. pylori* status before and after chemotherapy. Seven patients were seropositive before chemotherapy but became seronegative within 12 months. These seven patients had received antimicrobial therapy for bacterial infections during the treatment course and one also for *H. pylori* in the diagnostic phase.

### Oro-gastrointestinal symptoms during chemotherapy

Adverse events during 5-FU treatment were compared between patients who were *H. pylori*-seronegative and those who were *H. pylori-*seropositive at baseline and no statistically significant differences were observed (Table II). The worst oro-gastrointestinal toxicity was grade 3 or 4 in 54% of the seropositive and 62% in the seronegative patients (P=0.68). Functional dyspeptic symptoms (postprandial fullness, nausea, belching, early satiety, epigastric pain and burning) were present in 46% of the seropositive and in 48% of the seronegative patients (P=0.91). Digestive symptoms, including nausea, constipation and flatulence, were equally common (present in 43–86% of the patients) in both *H. pylori* groups. Severe mucositis, characterised as stomatitis or diarrhoea, was common (present in 81–93% of patients), but no differences between groups were observed.

Interaction between *H. pylori* status and chemotherapy regimen-related adverse events was observed only for diarrhoea of any grade (P=0.006), but no significant interaction was observed separately for grade 3–4 diarrhoea (P=0.110). In *H. pylori*-seropositive patients there was no significant difference between chemotherapy groups, as 65% in the bolus group vs. 85% in the infusion group had any grade of diarrhoea (P=0.16) and 24 vs. 20% had grade 3–4 diarrhoea (P=0.80). However, in seronegative patients any grade of diarrhoea was significantly more common in the bolus group (96 vs. 61%, P=0.018) and diarrhoea of grade 3–4 was more common in the bolus group (50 vs. 11%, P=0.015).

### Diagnostic delay from onset of symptoms to surgery for CRC

The most common symptoms of CRC present at diagnosis were bowel symptoms (including diarrhoea, constipation, alternating function or mucous faeces) in 53 (73%), blood in the stool in 36 (49%), functional dyspepsia in 26 (35%), anaemia in 13 (18%), occlusion/perforation in 10 (14%) and infectious symptoms in 10 (14%) patients.

**Figure 1:**
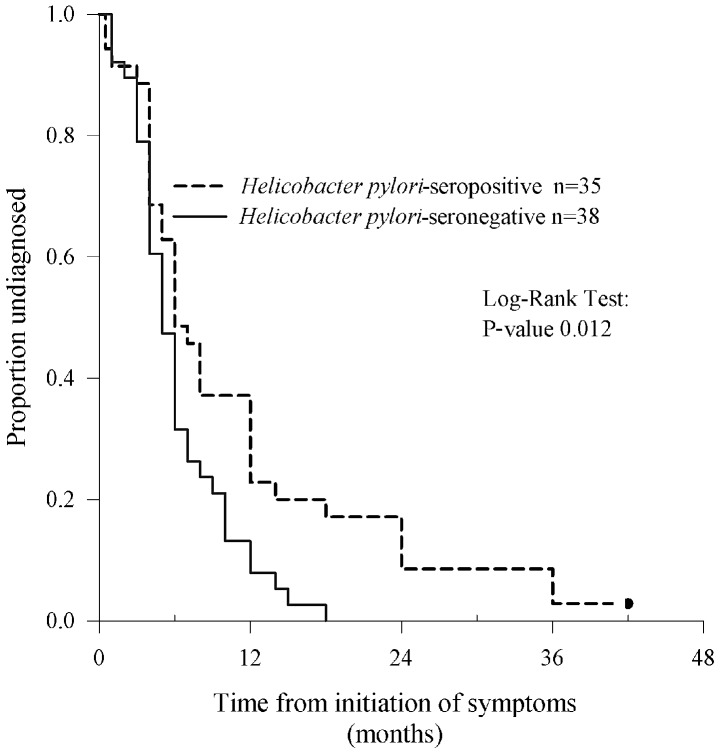
Time from initiation of symptoms to surgery in 73 colorectal cancer patients.

The median (inter quartile range, IQR) time from the onset of symptoms to CRC operation was 6 months (range, 4–11) in all 73 evaluable patients. The longest time from symptoms to surgery was 42 months. In the 35 *H. pylori-*seropositive patients, the delay was significantly longer than in the 38 seronegative patients; median 6 months (range, 4–12) vs. 5 months (range, 4–8) (P=0.012, Fig. 1). In six (17%) seropositive patients, but in none of the seronegative, the diagnostic delay was greater than 18 months.

Twenty six (35%) out of 73 patients who had functional dyspepsia at presentation (postprandial fullness, nausea, belching, early satiety, epigastric pain and burning) had significantly delayed diagnosis; median 7.5 months (range, 4–14) vs. 5 months (range, 2–8) (P=0.035). By comparison, in patients presenting with anaemia (*H. pylori*-seropositive, 26% vs. *H. pylori*-seronegative, 11%), bowel symptoms (77 vs. 68%), occlusion (11 vs. 16%), blood in the stool (43 vs. 55%) or infectious symptoms (14 vs. 13%) had no statistically significant diagnostic delays.

Lactose intolerance with oral lactose test was assessed in 77 (97%) of the patients. Fourteen (18%) patients, out of the 77 tested, were diagnosed with hypolactasia and 12 (16%) with borderline finding, whereas normolactasia was found in 51 (66%) patients. There was no correlation between hypolactasia and *H. pylori* seropositivity (P=0.20). Patients with hypolactasia, borderline or normolactasia did not differ significantly with respect to median diagnostic delay; 5 months (range, 4–10) vs. 5 months (range, 4–14) vs. 6 months (range, 4–10), respectively (log-rank test P=0.99).

### Disease-free and overall survival

**Figure 2:**
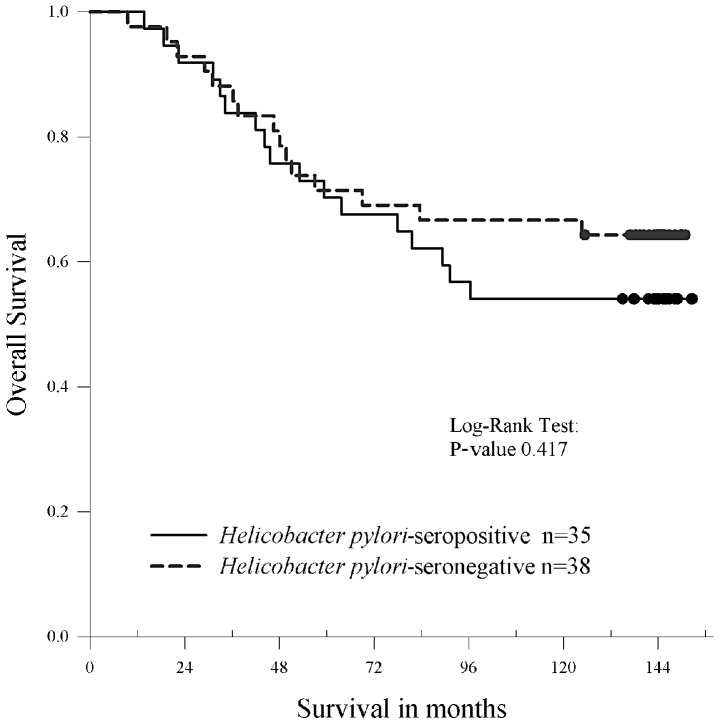
Overall survival in 73 stage II and III colorectal cancer patients divided at baseline by *Helicobacter pylori* seropositivity (n=35) and seronegativity (n=38).

The minimum follow-up time for the surviving 73 stage II and III patients was 120 months. At this 10-year time point the disease-free survival rate was 64% and the overall survival rate was 65%. Between groups, the disease free survival rate was 61% for *H. pylori-*seropositive patients vs. 67% for seronegative patients, and the overall survival rate was 61 vs. 69%. The differences in survival curves between seropositive and seronegative patients were statistically nonsignificant. Adjusted hazard ratios were 1.32 (95% CI, 0.64–2.72; P=0.453) and 1.34 (95% CI, 0.61–2.92; P=0.461) for disease-free and overall survival, respectively. The Kaplan-Meier survival curve for overall survival is shown in Fig. 2.

## Discussion

While the incidence of gastric cancer in western countries has declined along with the decreasing prevalence of *H. pylori* infection (6,19), CRC has become the third most common type of cancer in the western world and the second leading cause of cancer-associated mortality (20). This makes the impact of CRC and its treatment even more significant. In the present study, we investigated the possible role of *H. pylori* infection in CRC patients undergoing adjuvant chemotherapy. Although *H. pylori*-seropositive patients were not shown to develop gastrointestinal adverse events more often than the seronegative patients during treatment and there were no significant differences between the two groups in disease-free and overall survival rates, we demonstrated a significant delay in the diagnosis of CRC among the *H. pylori*-positive patients as compared with the *H. pylori*-negative patients. Of the different symptoms studied, functional dyspepsia was significantly associated with a delayed diagnosis of CRC.

Functional dyspepsia is a common complaint and often represents with a wide spectrum of upper abdominal symptoms, including postprandial fullness, nausea, belching, early satiety, epigastric pain and burning. Although the majority of *H. pylori* infected patients are asymptomatic, *H. pylori* is considered to be significant in functional dyspepsia. *H. pylori* eradication is thus far the best therapy available, and it provides long-term relief of symptoms in approximately one of 12 *H. pylori*-positive dyspepsia patients treated (5). Therefore screening of *H. pylori* and eradication treatment in infected dyspepsia patients are often considered as routine (4,21). In this study, patients with functional dyspepsia at diagnostic work-up and those who were *H. pylori*-seropositive at initiation of adjuvant chemotherapy, had a statistically significant delay in their diagnosis of CRC. Dyspeptic symptoms may first lead to gastroscopy or other interventions associated with *H. pylori* and, thus, result in postponed colonoscopy and delayed diagnosis of CRC. Colon cancer has previously been observed to mimic functional dyspepsia in one small retrospective patient series (22), which is in accordance with our present prospective study with functional dyspeptic symptoms misleading diagnostic work-up in CRC patients. The investigation of nonspecific symptoms, including functional dyspepsia and the subsequent diagnosis of *H. pylori,* may even postpone the diagnosis of malignancies other than CRC. We recently detected, in a large cohort of Finnish subjects who had received reimbursement for drugs used for *H. pylori* eradication, a significantly higher incidence of numerous different malignancies (including colon and rectum cancers) as compared with the average population (23).

Symptoms typically associated with CRC include changes in bowel habits, gastrointestinal bleeding leading to anaemia or blood in the stool, abdominal pain, weight loss and obstructive symptoms. In the diagnosis of CRC, the highest positive predictive values have been observed for rectal bleeding in combination with change in bowel habit or with perianal symptoms (3). In this study, when different symptoms were analysed in association with diagnostic delay, no significant differences were observed between *H. pylori-*seropositive and -seronegative patients.

Diagnostic CRC work-up is occasionally stopped when common complaints other than those likely associated with *H. pylori* have been diagnosed. Although *H. pylori* infection was the most common finding in our patient material, lactose intolerance was also frequent. The majority of the world’s population has hypolactasia but not all have symptoms and, thus, genetic and nutritional factors influence lactose tolerance. Although numerous typical symptoms of lactose intolerance, including abdominal pain, bloating, flatulence, diarrhoea, borborygmi, and in certain occasions nausea and vomiting (24), are the same as those in functional dyspepsia, there was no statistically significant diagnostic delay of CRC in patients with hypolactasia at baseline in our study.

The most common chemotherapy-associated side-effects are oro-gastrointestinal, including stomatitis, diarrhoea and nausea. These symptoms may have an extremely negative impact on quality of life during the treatment and may lead to dose reductions or, in the worst case, to early cessation of chemotherapy. We aimed to study whether *H. pylori* infection influences development of chemotherapy-associated side-effects in CRC patients. According to our results, *H. pylori-*seropositive patients tolerated the treatment equally well compared with *H. pylori*-seronegative patients. This is also in accordance with the findings that not all *H. pylori*-infected individuals possess symptoms (4). Although the number of patients in our study was relatively small, it appears unlikely that *H. pylori* infection would exaggerate oro-gastrointestinal toxicity during chemotherapy and, therefore, the screening or eradication of *H. pylori* in CRC patients prior to adjuvant treatment is unnecessary.

Among gastric cancer patients treated with surgery and adjuvant therapy, *H. pylori*-negative patients have an inferior outcome compared with *H. pylori*-positive individuals (25,26). The reason for this remains unexplained, but it may be associated with immunological aspects (25). Although the role of *H. pylori* in the carcinogenesis of CRC remains unclear, we evaluated the possible prognostic influence of *H. pylori* status in the survival of CRC patients treated with adjuvant chemotherapy. There were no significant differences in disease-free or overall survival between *H. pylori-*seropositive and *H. pylori*-seronegative CRC cohorts. Thus, the diagnostic delay observed in *H. pylori* positive patients did not become a significantly inferior curative outcome in this small series.

We conclude that *H. pylori* infection does not appear to increase toxicity during 5-FU-based adjuvant chemotherapy in operated CRC patients and, therefore, screening of *H. pylori* is not recommended in these patients prior to the start of the adjuvant chemotherapy. However, the most important finding in this study is that dyspeptic symptoms or the presence of *H. pylori* may lead to diagnostic delay of CRC. Although in this study we could not show inferior disease-free or overall survival in *H. pylori*-positive patients as compared with *H. pylori-*negative patients during the follow-up period of ≥10 years, it is important to emphasize that diagnostic CRC work-up should not be terminated due to diagnosis of chronic *H. pylori* infection.

## Figures and Tables

**Table I:** Patient characteristics in *Helicobacter pylori*-seronegative and -seropositive.

Characteristic	Total	*Helicobacter pylori-* seronegative	*Helicobacter pylori-*seropositive	P-value
Number, n (%)	79 (100)	42 (53)	37 (47)	
Median age, years (range)	60 (31–76)	58 (31–73)	60 (36–75)	0.89
Gender, n (%)				
Male	40 (51)	18 (43)	15 (41)	0.14
Female	39 (49)	24 (57)	22 (59)	
Tumour site, n (%)				
Colon	42 (53)	26 (62)	21 (57)	0.10
Rectum	37 (47)	16 (38)	16 (43)	
Tumour stage, n (%)				
II	19 (24)	9 (21)	10 (27)	0.68
III	54 (68)	31 (74)	23 (62)	
IV	6 (8)	2 (5)	4 (11)	
Chemotherapy, n (%)				
Bolus regimen	41 (52)	24 (57)	17 (46)	0.32
Infusion regimen	38 (48)	18 (43)	20 (54)	
Rectal radiotherapy, n (%)				
No	48 (61)	29 (69)	19 (51)	0.30
25/5 Gy	5 (6)	1 (2)	4 (11)	
45.0–50.4/1.8 Gy	26 (33)	12 (29)	14 (38)	
Lactose intolerance, n	77	40	37	
Hypolactasia, n (%)	14 (18)	6 (15)	8 (22)	0.20
Borderline, n (%)	12 (16)	9 (22)	3 (8)	
Normolactasia, n (%)	51 (66)	25 (63)	26 (70)	

**Table II:** Toxicity during chemotherapy in *Helicobacter pylori-*seronegative and -seropositive patients.

			*Helicobacter pylori*-seropositive vs. -seronegative patients		
			
Toxicity/grade	Seronegative patients (n=42), n (%)	Seropositive patients (n=37), n (%)	OR^a^	95% CI	P-value
Worst oro-gastro-intestinal toxicity					
None	0 (0)	0 (0)	0.82	0.32–2.11	0.68
Grade 1–2	16 (38)	17 (46)			
Grade 3–4	26 (62)	20 (54)			
Stomatitis					
None	3 (7)	6 (16)	0.65	0.23–1.86	0.43
Grade 1–2	24 (57)	22 (60)			
Grade 3–4	15 (36)	9 (24)			
Functional dyspepsia					
None	22 (52)	20 (54)	0.95	0.39–2.32	0.91
Grade 1–2	19 (45)	16 (43)			
Grade 3–4	1 (2)	1 (3)			
Diarrhoea^b^					
None	8 (19)	9 (24)	0.61	0.21–1.73	0.35
Grade 1–2	20 (48)	20 (54)			
Grade 3–4	14 (33)	8 (22)			
Constipation					
None	23 (55)	21 (57)	0.94	0.38–2.30	0.89
Grade 1–2	19 (45)	16 (43)			
Grade 3–4	0 (0)	0 (0)			
Flatulence					
None	14 (33)	17 (46)	0.55	0.22–1.40	0.21
Grade 1–2	27 (64)	20 (54)			
Grade 3–4	1 (2)	0 (0)			
Nausea					
None	6 (14)	12 (32)	1.52	0.37–6.21	0.56
Grade 1–2	32 (76)	20 (54)			
Grade 3–4	4 (10)	5 (14)			

a The incidence of grade 3–4 toxicity was compared between *Helicobacter pylori*-seropositive and -seronegative patients. In functional dyspepsia, constipation and flatulence the incidence of grade 1–4 toxicity was analysed. The ORs are adjusted for chemotherapy group (infusion regimen vs. bolus regimen) in binary logistic regression analysis.

b P=0.110 for interaction between *Helicobacter pylori* seropositivity and chemotherapy treatment. OR, odds ratio; CI, confidence interval.
